# Comparison between the perceptions of family members and health professionals regarding a flexible visitation model in an adult intensive care unit: a cross-sectional study

**DOI:** 10.5935/0103-507X.20220114-en

**Published:** 2022

**Authors:** Cláudia Severgnini Eugênio, Tarissa da Silva Ribeiro Haack, Cassiano Teixeira, Regis Goulart Rosa, Emiliane Nogueira de Souza

**Affiliations:** 1 Universidade Federal de Ciências da Saúde de Porto Alegre - Porto Alegre (RS), Brazil.; 2 Hospital Humaniza - NotreDame Intermedica - Porto Alegre (RS), Brazil.; 3 Research Projects Office, Hospital Moinhos de Vento - Porto Alegre (RS), Brazil.

**Keywords:** Critical care, Intensive care units/organization & administration, Visitors to patients, Family, Patient care team, Perception

## Abstract

**Objective:**

To compare the perceptions of patients’ relatives with the perceptions of health professionals regarding a flexible visitation model in intensive care units.

**Methods:**

Cross-sectional study. This study was carried out with patients’ relatives and members of the care team of a clinical-surgical intensive care unit with a flexible visitation model (12 hours/day) from September to December 2018. The evaluation of the flexible visitation policy was carried out through an open visitation instrument composed of 22 questions divided into three domains (evaluation of family stress, provision of information, and interference in the work of the team).

**Results:**

Ninety-five accompanying relatives and 95 members of the care team were analyzed. The perceptions of relatives regarding the decrease in anxiety and stress with flexible visitation was higher than the perceptions of the team (91.6% *versus* 58.9%, p < 0.001), and the family also had a more positive perception regarding the provision of information (86.3% *versus* 64.2%, p < 0.001). The care team believed that the presence of the relative made it difficult to provide care to the patient and caused work interruptions (46.3% *versus* 6.3%, p < 0.001).

**Conclusion:**

Family members and staff-intensive care unit teams have different perceptions about flexible visits in the intensive care unit. However, a positive view regarding the perception of decreased anxiety and stress among the family members and greater information and contributions to patient recovery predominates.

## INTRODUCTION

The critical care setting may expose family members to a variety of stressors, such as problems with communication, uncertainty about patient survival or rehabilitation, and lack of support for shared decisions.^(1)^ Traditionally, around the world, visitation to intensive care unit (ICU) patients occurs at restricted times based on the theoretical risk of increased physiological stress, the damage to the organization of critical care, and the increased risk of infectious complications caused by a flexible visitation policy.^(2-4)^ However, many ICUs are shifting their restrictive visitation policy to open or flexible visitation to foster patient-centered care and to improve family and patient satisfaction.^(5-7)^ Previous studies have shown that symptoms of anxiety and depression decrease with flexible visits and satisfaction increases. In addition, there has been no increase in burnout within the care team.^(7)^

Nevertheless, many professionals continue to resist and believe that the presence of the relative can lead to a greater workload for the care team and to greater disorganization of care to patients.^(8,9)^ Knowledge of the points of convergence and divergence of professionals and relatives regarding the flexible visit can contribute to optimizing a model that pleases patients, families, and staff, since the main goal is the recovery and care of the patient in the intensive care setting.^(10-13)^ The evaluation of a flexible visitation policy in the ICU, through the perception of the care team and the accompanying family members, is a way to improve this practice and improve the development of care processes, guaranteeing humanized care. In addition, it provides a context in which to foster an environment of learning and trust for everyone involved in the hospitalization process.

Thus, the objective of the present study was to compare the perceptions of patients’ relatives with the perceptions of health professionals with regard to a flexible family visitation model in the ICU.

## METHODS

### Population studied

A cross-sectional study was performed in an adult clinical and surgical ICU of a 56-bed, tertiary private hospital with a flexible family visitation in Southern Brazil.

In this ICU, each nursing technician serves a maximum of two patients, while nurses, physiotherapists, and doctors serve up to ten patients. This visitation model in place since 2015 allows up to two relatives to remain at the patient’s bedside for the period from 9:00 a.m. to 9:00 p.m. For the family member to have the right to continue to stay with the patient, it is necessary for this family member to participate in an informational meeting on good practices of ICU visitation. This meeting takes place daily, and aspects related to the operation of the unit, the care that critically ill patients will receive, the infection control measures, and the rights and duties of the ICU visitor are explained. In addition, the accompanying family member must agree to sign a commitment term provided after passing on the information, which includes the companion’s rights and duties. A physician, who is not necessarily an intensivist, is responsible for the patient. This doctor shares decisions about patient care with the care team and talks to family members daily. Despite the care team not having the responsibility to pass on information about the patient’s health status, in many instances, they end up clarifying family members’ concerns at the bedside. Data collection was performed by convenience and was carried out from September to December 2018.

Inclusion criteria for accompanying family members were family members of hospitalized patients of both sexes (parents, children, siblings, or spouses) who were over 18 years of age, who remained at the patient’s bedside for a period longer than two hours a day, and whose patient had been hospitalized for more than 48 hours in the unit, regardless of the reason for the hospitalization. Caregivers assigned by the relative responsible for the patient were also included. Family members and caregivers who had cognitive or visual deficits in completing the questionnaire were excluded from the study. ICU care team members (nurses, nursing technicians, physiotherapists, nutritionists, psychologists, and routine physicians) were also included in the study according to the following inclusion criteria: they were part of the ICU staff; they had been working in the unit for at least three months, and they had exposure to flexible visitation for more than two hours a day. A questionnaire was administered to all ICU workers who agreed to participate in the study. There was no exclusion of questionnaires.

The institutional review board reviewed and approved this study (CAAE nº 54454016.5.0000.5345).

### Data collection

The assessment of the flexible visitation policy by the care team was carried out through the evaluation instrument of open visitation, which is composed of 22 questions divided into three domains (i.e., evaluation of family stress, provision of information, and interference in the work of the team).^(11)^ All questions had Likert scale answers: never (1); occasionally (2); often (3); and always (4), except for questions 20, 21, and 22, for which there were three possible answers: yes (1); no (2); or I do not know (3). Questions Q3, Q4, Q9, Q10, Q11, Q12, Q13, Q14, and Q15 had their answers inversely coded. The first 19 questions were grouped as negative (never/occasionally) or positive (often/always) for better distribution of results.

Subjects who were accompanying the hospitalized relative were invited to participate in the study and, after acceptance, signed the informed consent form. The questionnaires were given to the individuals who answered in a private place, near or inside the ICU, and afterward were left in a reserved place in the research room. The same instrument was used to evaluate the flexible visitation policy with accompanying family members, with adaptations in the questions for better comprehension purposes. Both instruments were self-administered, and the questionnaire completion time was approximately 30 minutes. Sociodemographic variables were also collected from family members and the care team.

### Sample size

The sample calculation for the care team members was based on a previous study which used an open-visitation questionnaire.^(11)^ Considering positive evaluation answers approximately 44.8% in the care team, with a 5% error and a significance of 5%, based on the contingent of professionals working in the sector, 95 participants were needed. The same number of participants was chosen for the family group.

### Statistical analysis

The data were analyzed in a descriptive and analytical way by the Statistical Package for Social Sciences software (SPSS), version 21.0. Categorical variables are presented as absolute (n) and relative (%) numbers. Continuous variables are presented as the mean and standard deviation (SD). Categorical variables were compared using the chi-square test. The comparison of the responses between the groups was performed by the Mann-Whitney test. A two-tailed p < 0.005 was considered statistically significant.

## RESULTS

A total of 95 family members of hospitalized ICU patients and 95 members of the care team were included. The average time of professional exercise of the care team members was a median of 3 (1 - 5) years, with a mean age of 32 years (SD = 6), and the average time of experience with open visits was two years.

The mean age of the accompanying family members was 51 years (SD = 12). Regarding the neurological status of the patients, 63 (66.3%) were conscious/verbalizing. The other demographic characteristics are described in table 1.

**Table 1:** Characterization of the study subjects

Variable	
Health care team	
Women	78 (82.1)
Profession	
Nursing technicians	57 (60)
Nurses	19 (20)
Physicians	11 (11.6)
Other	8 (8.4)
Working shift	
Day	69 (72.6)
Night	26 (27.4)
Average time working (years)	3 (1 - 5)
Relatives and caregivers	
Women	68 (71.6)
Education	
College	65 (68.4)
High School	23(24.2)
Elementary School	7 (7.4)
Relation	
Son (daughter)	48 (50.5)
Spouse	31 (32.7)
Caregiver	16 (16.8)
Occupational status	
Active	71 (74.7)
Retired	24 (25.3)

Results expressed as the n (%) or median (25% - 75% percentile).

### Comparison of the perceptions of flexible visitation

Accompanying relatives have a more positive view concerning the flexible visitation model when compared to the health care team. Table 2 shows the percent of responses grouped as negative (never/occasionally) or positive (frequently/always) for a better distribution of the results. Questions Q3, Q4, Q9, Q10, Q11, Q12, Q13, Q14, and Q15 had their answers inversely coded. All other answers differed significantly, except for answers 12 and 18.

**Table 2:** Comparison of the responses provided by the group of accompanying relatives and the health care team

	Accompanying relatives		Health care team		p value
	Negative	Positive	Negative	Positive	
Q1 - Do you think flexible visitation helps patient recovery?	7 (7.4)	88 (92.6)	23 (24.2)	72 (75.8)	< 0.001
Q2 - Do you think flexible visitation decreases stress and anxiety in patients?	7 (7.4)	88 (92.6)	35 (36.8)	60 (63.2)	< 0.001
Q3 - Do you think flexible visitation makes it more difficult to provide care to the patient?	11 (11.6)	84 (88.4)	27 (28.4)	68 (71.6)	< 0.001
Q4 - Do you think flexible visitation interferes with patient privacy?	5 (5.3)	90 (94.7)	18 (18.9)	77 (81.1)	< 0.001
Q5 - Do you think flexible visitation decreases anxiety and stress in the family?	8 (8.4)	87 (91.6)	39 (41.1)	56 (58.9)	< 0.001
Q6 - Do you think flexible visitation increases family confidence?	24 (25.3)	71 (74.7)	29 (30.6)	66 (69.4)	< 0.001
Q7 - Do you think the increase in visitation time contributes to the satisfaction of the family in relation to the team?	6 (6.4)	89 (93.7)	18 (19)	77 (81)	< 0.001
Q8 - Do you think flexible visitation allows for the family to have more information about the patient?	13 (13.7)	82 (86.3)	34 (35.8)	61 (64.2)	< 0.001
Q9 - Do you think flexible visitation forces the family to stay with the patient?	6 (6.3)	89 (93.7)	25 (26.3)	70 (73.7)	< 0.001
Q10 - Do you think flexible visitation impairs the organization of the health care provided to the patient?	3 (3.2)	92 (96.8)	18 (18.9)	77 (81.1)	< 0.001
Q11 - Do you think the work of intensive care unit professionals suffers more interruptions due to the flexible visitation?	6 (6.3)	89 (93.7)	44 (46.3)	51 (53.7)	< 0.001
Q12 - Do you think flexible visitation interferes in the work priorities of intensive care unit professionals?	16 (16.8)	79 (83.1)	21 (22.1)	74 (77.9)	0.47
Q13 - Do you think flexible visitation leads to a delay in the analysis and performance of procedures for the patients?	3 (3.2)	92 (96.8)	23 (24.2)	72 (75.8)	< 0.001
Q14 - Do you think professionals feel uncomfortable when they examine patients in the presence of the family?	2 (2.2)	93 (97.8)	25 (26.3)	70 (73.7)	< 0.001
Q15 - Do you think professionals feel troubled by the prolonged presence of the patient’s family?	7 (7.4)	88 (92.6)	14 (14.8)	81 (85.2)	< 0.001
Q16 - Do you think flexible visitation contributes to a change in work attitudes in the intensive care unit?	38 (40)	57 (60)	74 (77.9)	21 (22.1)	< 0.001
Q17 - Do you think flexible visitation helps the family to feel responsible for the care of the patient?	3 9(41.1)	56 (58.9)	55 (57.9)	40 (42.1)	< 0.001
Q18 - Do you think intensive care unit visitation must be changed in the case of conflict or by request of the patient?	29 (30.5)	66 (69.5)	21 (22.1)	74 (77.9)	0.35
Q19 - Do you think intensive care unit visitation must be changed in special cases, such as end of life?	36 (37.9)	59 (62.1)	14 (14.8)	81 (85.2)	< 0.001

Results expressed as the n (%).

Among the questions that presented the greatest difference in the responses were those related to the fact that the family member made it more difficult to provide care to the patient (Q3), the decrease in anxiety and stress in the family (Q5), the provision of more information while being with the patient (Q8), interruption in the team’s work (Q11), discomfort in the health team (Q14), changes in the team’s attitudes (Q16), and the fact that the flexible visitation is changed in special cases (Q19).

In the research ICU, 98.9% of accompanying family members *versus* 84.2% of the assisting team agreed to having access to extended visits in the case of a family member, with 90.5% of the family considering the team trained in communicating; however, only 31.6% of the team had this perception, and 76.8% of the care team would like to receive communication training. Other data in relation to questions 20, 21, and 22 are presented in table 3.

**Table 3:** Comparison of the responses provided by the group of accompanying relatives and those of the health care team

Question	Family	Question	Team
Q20 - Do you agree in having access to flexible visitation in the hospitalization of your relative?	98.9	Q20 - If you or your relatives needed hospitalization in the intensive care unit, would you like to have access to flexible visitation?	84.2
Q21 - Do you consider the team as trained in communicating with relatives of patients in the intensive care unit?	90.5	Q21 - Have you attended any trainings related to how to communicate with family members at the intensive care unit?	31.6
Q22 - Do you believe the team needs training to improve communication skills with patients' relatives in the intensive care unit with flexible visitation?	22.1	Q22 - Would you like to receive training to improve your ability to communicate with the intensive care unit patient's family with flexible visitation?	76.8

Results expressed as the %.

## DISCUSSION

In this study, we observed that both groups agreed that the companion’s stay at the bedside is a factor that benefits to the patient’s recovery, alleviates family suffering, reduces the perception of anxiety and stress in patients and families, allows for more information about the patient’s clinical condition, and increases family satisfaction. There were differences in responses regarding interference in the work of the care team, changes in attitudes in the care team’s work, and team training for the orientation of the family with the flexibility of visitation in the ICU.

The ICU visits study by Rosa et al. showed that a flexible family visitation policy supported by family education did not significantly reduce the incidence of delirium among patients compared with standard restricted visitation, contrary to previous studies. However, this study did show a reduction in symptoms of anxiety and depression and better satisfaction among family members.^(7)^ It is known that the presence of family members in ICU settings for a longer period of time improves patient and family satisfaction and reduces anxiety and delirium in patients; however, this may be associated with an increased risk of burnout among ICU professionals.^(14-19)^ Some professionals perceive that the presence of families disorganizes the care provided to the patient and presents greater workload and occupational stress.^(10,11)^ However, studies indicate that the increased presence of relatives in the ICU can contribute to a better understanding of the patient’s needs and, consequently, to the quality of the care provided.^(18,19)^ The better outcomes observed with flexible visitation may be mediated by better communication, proximity to the patient, reassurance, and support.^(7)^

Regarding the training of the care team to communicate with family members, most professionals answered that they did not receive training and that they would like to improve their ability to communicate, whereas family members considered the team trained to communicate with family members. It is noteworthy that the training of the care team to receive and inform these family members is of fundamental importance, since the daily work of the team, especially nursing in many situations, requires professional interaction with patients and their families.^(20,21)^

A study that aimed to evaluate the team’s perception of open visitation to ICUs revealed that, of the 106 participants, 79.2% of ICU team members had difficulties communicating with families, and 84% reported a desire to acquire good communication skills.^(10)^ Multiprofessional team meetings with family members in the first 24 - 48 hours after patient admission may be one of the alternatives to improve communication techniques, establish rules and clarify doubts, set goals to alleviate stress and anxiety of the family members, and establish agreements of the rights and duties of the companions during the period of hospitalization.^(22,23)^

The presence of the family next to the patient allows them to be active agents of care; that is, the family should be understood as an important ally of the team and can act as a resource through which the patient can reaffirm and, often, recover his or her own participation in the treatment.^(24,25)^ In this sense, patient-centered care begins to be an ethical issue that should be discussed with health services, and from this, adequate policies must be developed to support flexible visitation in intensive health care services.

The strengths of our study are that these data can be taken into consideration in the planning and implementation of flexible visitation programs or policies in the setting of intensive care and in discussions among care teams about the presence of relatives at the bedside in new spaces of social interactions. We suggest future studies in several centers that are able to evaluate patient- and family-centered care and the benefits brought about by flexible visitation in adult ICUs.

This study has some limitations. First, the research was conducted at a single center. Second, as it is a self-administered instrument, some questions may have unreliable answers due to the lack of understanding of the question itself. In addition, the family visiting hours were not verified during the research, a fact that may impact the participants’ responses. Another limitation is that decisions on giving information to family members regarding the health status of patients are the responsibility of the attending physician, who is often not an intensivist and spends little time with the patient during treatment. Thus, there may be a favorable bias toward the open visit on the part of family members. In many ICUs, the responsibility for all care and for communication with family members is the intensivist. In these cases, open visitation can cause more strain on the care team as well as interfere with the perception of the family’s presence in the ICU for a prolonged period.

## CONCLUSION

Both groups of family and care staff are in favor of the flexible visitation policy. However, family members offered a more positive evaluation than did members of the care team. Among the main benefits, we highlight aspects, such as the perception of a decrease in anxiety and stress among accompanying family members and a contribution to the recovery of the patient. Among the negative aspects, we report interference in the work of the health care team.
